# Neuroscience of Exercise: Neuroplasticity and Its Behavioral Consequences

**DOI:** 10.1155/2016/3643879

**Published:** 2016-10-13

**Authors:** Henning Budde, Mirko Wegner, Hideaki Soya, Claudia Voelcker-Rehage, Terry McMorris

**Affiliations:** ^1^Faculty of Human Sciences, Medical School Hamburg, Kaiserkai 1, 20457 Hamburg, Germany; ^2^Lithuanian Sports University, Kaunas, Lithuania; ^3^Physical Activity, Physical Education, Health and Sport Research Centre (PAPESH), Sports Science Department, School of Science and Engineering, Reykjavik University, Reykjavik, Iceland; ^4^Institute of Sport Science, University of Bern, Bremgartenstrasse 145, 3012 Bern, Switzerland; ^5^Laboratory of Exercise Biochemistry and Neuroendocrinology, Faculty of Health & Sport Sciences, University of Tsukuba, Tsukuba, Japan; ^6^Institute of Human Movement Science and Health, Faculty of Behavioral and Social Sciences, Technische Universität Chemnitz, Chemnitz, Germany; ^7^Institute of Sport, University of Chichester, Chichester, UK

The human brain adapts to changing demands by altering its functional and structural properties (neuroplasticity) which results in learning and acquiring skills. Convergent evidence from both human and animal studies suggests that enhanced physical exercise facilitates neuroplasticity of certain brain structures and as a result cognitive functions [1] as well as affective [2] and behavioral responses [3]. This special issue is being proposed at a very challenging time. There is evidence linking increased physical exercise with an enhancement of neurogenesis, synaptogenesis, angiogenesis, and the release of neurotrophins as well as neuroendocrinological changes, which are associated with benefits in cognitive and affective as well as behavioral functioning (such as fine motor functioning).

The body-mind connection has recaptured scientific interest in recent years with more than a dozen of academic books dedicated to the topic. This reflects the growth in interest.* Physical activity* refers to body movement that leads to energy expenditure and is initiated by skeletal muscles [4, 5].* Physical exercise* or exercise has been previously defined as a disturbance of homeostasis through muscle activity resulting in movement and increased energy expenditure [6]. The critical difference between both terms refers to the planned and structured nature of exercise [4].

This present special issue has one primary aim: enhancing our understanding of the neurobiological mechanisms of physical exercise, which result in different experiential and behavioral consequences. It includes six original contributions from international research groups. The researchers used different methods including brain scans, EEG recordings, and blood as well as saliva sampling to investigate the exercise-induced brain activity and volume changes in different brain areas including frontal and central regions of the brain, the hippocampus, cerebellum, and the motor cortex. The effects of a variety of physical exercises like dancing, playing handball, walking, or cycle ergometry were assessed. The outcome variables referred to cognitive (memory consolidation) and motor performance measures.

In their study “Senior Dance Experience, Cognitive Performance, and Brain Volume in Older Women,” C. Niemann et al. (2016) tested the association of long-term dance experience with cognitive performance and gray matter brain volume in older women aged 65 to 82 years. The German research group compared nonprofessional senior dancers (*n* = 28) with nonsedentary control group participants without any dancing experience (*n* = 29), who were similar in age, education, IQ score, lifestyle and health factors, and fitness level. Neither in the four tested cognitive domains (executive control, perceptual speed, episodic memory, and long-term memory) nor in brain volume (VBM whole-brain analysis and region-of-interest analysis of the hippocampus) were differences observed. Results indicated that moderate dancing activity (1-2 times per week, on average) has no additional effects on gray matter volume and cognitive functioning when a certain lifestyle or physical activity and fitness level is reached.

In the study by R. Thomas et al. entitled “Acute Exercise and Motor Memory Consolidation: The Role of Exercise Timing,” the Danish-US-American-Canadian research group investigated the effects of three different temporal placements of high-intensity exercise following visuomotor skill acquisition on the retention of motor memory in 48 young (24.0 ± 2.5 yrs), healthy male subjects. These were randomly assigned to one of four groups either performing a high-intensity (90% maximum power output) cycle-ergometer exercise bout for 20 min (EX90), 1 h (EX90+1), and 2 h (EX90+2) after acquisition or rested (CON). Retention tests were performed at day one (R1) and day seven (R7) after testing. At R1 changes in performance scores from postacquisition were greater for EX90 than CON and EX90+2. At R7 changes in performance scores for EX90, EX90+1, and EX90+2 were higher than CON. Changes for EX90 at R7 were greater than EX90+2. Exercise-induced improvements in procedural memory diminish as the temporal proximity of exercise from acquisition is increased. Timing of exercise following motor practice is important for motor memory consolidation.

A Swiss study (J. Meier et al., 2016) entitled “Differences in Cortical Representation and Structural Connectivity of Hands and Feet between Professional Handball players and Ballet Dancers” aimed at investigating sport-specific adaptations in professional handball players versus ballet dancers. The research team focused on the primary motor and somatosensory gray matter (GM) representation of hands and feet using voxel-based morphometry as well as on fractional anisotropy (FA) of the corticospinal tract by means of diffusion tensor imaging-based fibre tractography. As predicted, GM volume was increased in hand areas of handball players, whereas ballet dancers showed increased GM volume in foot areas. Compared to handball players, ballet dancers showed decreased FA in both fibres connecting the foot and hand areas, and they showed lower FA in fibres connecting the foot compared to their hand areas, whereas handball players showed lower FA in fibres connecting the hand compared to their foot areas. The results suggest that structural adaptations are sport specific and are manifested in brain regions associated with the neural processing of sport-specific skills.

The study “Neural Correlates of Dual-Task Walking: Effects of Cognitive Versus Motor Interference in Young Adults” by R. Beurskens et al. (2016), a German research group, aimed to gather more information regarding the underlying neural correlates of single and dual-task walking. They had 15 young adults (23.9 ± 2.6 years) walking while concurrently performing a cognitive (CI) or a motor interference (MI) task. Simultaneously, neural activation in frontal, central, and parietal brain areas was registered using a mobile EEG system. Results showed that the MI task but not the CI task affected walking performance in terms of significantly decreased gait velocity, decreased stride length, significantly increased stride time, and increased tempo-spatial variability. Average activity in alpha and beta frequencies was significantly modulated during both CI and MI walking conditions in frontal and central brain regions, indicating an increased cognitive load during dual-task walking. Their results suggest that impaired motor performance during dual-task walking is mirrored in neural activation patterns of the brain. This finding is in line with established cognitive theories arguing that dual-task situations overstrain cognitive capabilities resulting in motor performance decrements.

Another interesting German study by K. Hötting et al. (2016) entitled “The Effects of Acute Physical Exercise on Memory, Peripheral BDNF, and Cortisol in Young Adults” aims to assess the effects of a single bout of physical exercise on memory consolidation and the possible underlying neuroendocrinological mechanisms in young adults (aged 22 years). Participants encoded a list of German-Polish vocabulary before exercising for 30 min at either high intensity or low intensity or relaxing. Retention of the vocabulary was assessed 20 minutes after the intervention as well as 24 hours later. Serum BDNF and salivary cortisol were measured at baseline, after learning and after the intervention. The high-intensity exercise group showed an increase in BDNF and cortisol after exercising compared to baseline. Exercise after learning did not enhance the absolute number of recalled words. Participants of the high-intensity exercise group, however, forgot less vocabulary than the relaxing group 24 hours after learning. There was no robust relationship between memory scores and the increase in BDNF and cortisol, respectively, suggesting that further parameters have to be taken into account to explain the effects of exercise on memory in humans.

The Canadian study “Promoting Motor Cortical Plasticity with Acute Aerobic Exercise: A Role for Cerebellar Circuits” by C. S. Mang et al. (2016) investigated the effect of acute aerobic exercise on cerebellar circuits and their potential contribution to altered M1 plasticity in healthy individuals (age: 24.8 ± 4.1 years). In experiment 1, acute aerobic exercise reduced cerebellar inhibition (CBI), elicited by dual-coil paired pulse transcranial magnetic stimulation. In experiment 2, they evaluated the facilitatory effects of aerobic exercise with response to paired associative stimulation, delivered with a 25 ms (PAS25) or 21 ms (PAS21) interstimulus interval. Increased M1 excitability evoked by PAS25, but not PAS21, relies on transcerebellar sensory pathways. The magnitude of the aerobic exercise effect on PAS response was not significantly different between PAS protocols; however, planned comparisons indicated that, relative to a period of rest, acute aerobic exercise enhanced the excitatory response to PAS25 but not PAS21. Thus, the results of these planned comparisons indirectly provide modest evidence that modulation of cerebellar circuits may contribute to exercise-induced increases in M1 plasticity. The findings have implications for developing aerobic exercise strategies to “prime” M1 plasticity for enhanced motor skill learning in applied settings.

The idea behind this special issue on the neuroscience of exercise with regard to neuroplasticity and its behavioral consequences was to foster the understanding of the link between exercise or physical activity and neuroscience. In this issue we have drawn together researchers from different disciplines, for example, brain science, cognitive psychology, neuroscience, and exercise science as well as psychophysiology, to present a state-of-the-art summary of what is known about the neurobiological mechanisms of physical exercise which result in behavioral consequences. The results suggest that physical exercise may trigger neuroplasticity and, thereby, enhances an individual's capacity to respond to new demands with behavioral alterations. We hope that the readers interested in the neuroscience of exercise perceive the findings presented as an update of the current literature and as exciting as we think they are. We further hope that the research presented encourages new studies on the neuroscience of exercise in the future.

*Henning Budde* *Henning Budde* *Mirko Wegner* *Mirko Wegner* *Hideaki Soya* *Hideaki Soya* *Claudia Voelcker-Rehage* *Claudia Voelcker-Rehage* *Terry McMorris* *Terry McMorris*
